# Identification of Differential MicroRNAs in Cerebrospinal Fluid and Serum of Patients with Major Depressive Disorder

**DOI:** 10.1371/journal.pone.0121975

**Published:** 2015-03-12

**Authors:** Yunqiang Wan, Yuanhui Liu, Xiaobin Wang, Jiali Wu, Kezhi Liu, Jun Zhou, Li Liu, Chunxiang Zhang

**Affiliations:** 1 Department of Anesthesiology and Psychiatry, Affiliated Hospital of Luzhou Medical College, Luzhou, Sichuan Province, China; 2 Department of Psychiatry, Affiliated Hospital of Luzhou Medical College, Luzhou, Sichuan Province, China; 3 Department of Pharmacology, Rush Medical College, Rush University, Chicago, IL, United States of America; National University of Singapore, SINGAPORE

## Abstract

Major depression is a debilitating disease. To date, the development of biomarkers of major depressive disorder (MDD) remains a challenge. Recently, alterations in the expression of microRNAs (miRNAs) from post-mortem brain tissue and peripheral blood have been linked to MDD. The goals of this study were to detect the differential miRNAs in cerebrospinal fluid (CSF) and serum of MDD patients. First, the relative expression levels of 179 miRNAs (relative high levels in serum) were analyzed by miRNA PCR Panel in the CSF of MDD patients. Then, the differentially altered miRNAs from CSF were further assessed by qRT-PCR in the serum of the same patients. Finally, the serum differentially altered miRNAs were further validated by qRT-PCR in the serum of another MDD patients. The CSF-results indicated that 11 miRNAs in MDD patients were significantly higher than these in control subjects, and 5 miRNAs were significantly lower than these in control subjects. The serum-results from the same patients showed that 3 miRNAs (miR-221-3p, miR-34a-5p, and let-7d-3p) of the 11 miRNAs were significantly higher than these in control subjects, and 1 miRNA (miR-451a) of 5 miRNAs was significantly lower than these in control subjects. The up-regulation of miR-221-3p, miR-34a-5p, let-7d-3p and down-regulation of miR-451a was further validated in another 32 MDD patients. ROC analysis showed that the area under curve of let-7d-3p, miR-34a-5p, miR-221-3p and miR-451a was 0.94, 0.98, 0.97 and 0.94, with specificity of 90.48%, 95.24%, 90.48% and 90.48%, and sensitivity of 93.75%, 96.88%, 90.63% and 84.85%, respectively. In addition, target gene prediction found that the altered miRNAs are involved in affecting some important genes and pathway related to MDD. Our results suggested that differentially altered miRNAs in CSF might be involved in MDD, and serum miR-221-3p, miR-34a-5p, let-7d-3p, and miR-451a might be able to serve as biomarkers for MDD.

## Introduction

Major depressive disorder (MDD) is a common chronic mood illness, resulting in heavy social and economic burdens [1, 2]. However, current diagnosis of MDD is mainly based on the patients' description of symptoms, assessment of mental status and evaluation of clinical behaviors, which increase the chance of misdiagnosis. There is no reliable biological marker from blood or cerebrospinal fluid that can be used for the diagnosis of MDD.

MicroRNAs (miRNAs) are small noncoding RNA molecules that regulate the stability and/or the translational efficiency of target messenger RNAs [3] and can influence the activity of approximately 50% or more of all protein-coding genes in mammals [4, 5], and several studies have reported that miRNAs are very important regulatory factors in the normal developmental, physiological and disease states, including cancer, mental disorders and cardiovascular diseases[6–8]. Recently, alterations in miRNAs expression from post-mortem brain tissues and peripheral blood samples have been linked to MDD [9,10].

However, unlike tumor tissue, some tissues such as from the heart and brain are not easy to obtain in clinical practice. Interestingly, recent studies have found that the diseased tissues of heart or brain can release miRNAs into the circulatory blood or CSF, and these miRNAs may be new biological markers of cardiovascular and cerebral diseases [11,12].

CSF is separated from the blood circulation by the blood brain barrier; therefore, the change of miRNAs in CSF might better reflect the alteration of miRNAs from the diseased tissues of brain than blood [13]. Unfortunately, the change in CSF miRNAs in MDD is unclear, and it is very difficult to draw CSF from MDD patients via lumber puncture. Therefore, the detection of serum miRNAs is easier and convenient than CSF. The goals of this study were to detect the candidate miRNAs in cerebrospinal fluid of MDD patients and to investigate these candidate miRNAs in serum from MDD patients further to provide information about miRNAs as specific biomarkers for the diagnosis of MDD.

## Methods

### Patients

The protocols of this study and the procedures employed for sample collection were approved by the Ethical Committee of Luzhou Medical College (Ky2012044). Informed written consent was obtained from all participants. Subjects with major depression were diagnosed according to either the ICD-10 or DSM IV criteria with a total score of 15 or higher on the 24-item HDRS. The exclusion criteria were as follows: a personal history of bipolar affective disorder, schizophrenia, mood incongruent psychotic symptoms, primary substance abuse or primary organic disease, current treatment with antidepressants or contraindication of lumbar puncture. The inclusion criteria of control subjects were as follows: no psychiatric disorders according to either the ICD-10 or DSM IV criteria, no serious systemic diseases, no complications and tumor, and these control patients also matched the above the exclusion criteria.

### Samples preparation and RNA Extraction

First, CSF samples of 6 depressed and 6 control patients were collected by diagnostic lumbar puncture or spinal anesthesia due to elective surgery (renal calculus, bladder stones), the blood samples of these patients were collected at the same time. Subsequently, the blood samples of another 32 MDD patients and 21 healthy individuals were collected. CSF samples were centrifuged (500×*g*, 10 min, room temperature) and blood samples were centrifuged (3000×*g*, 10 min, room temperature) within 60 min after collection to remove cells and debris and were stored at -80°C until further analysis. Hemorrhagic CSF samples were not included. Total RNA was extracted using with Trizol (Invitrogen, Carlsbad, CA, USA) based isolation kit (Shanghai Kangchen Biotechnology Company, Shanghai, China) according to the manufacturer’s protocol. The concentration and purity of RNA were analyzed by the NanoDrop-1000 (Thermo Scientific, Waltham, MA, USA).

### Real-time PCR

Approximately 20–25 ng RNA was retro-transcribed into cDNA with the MicroRNA Reverse Transcription Kit and the RT Primer Pools (Exiqon, Danmark) according to the manufacturer’s protocol. First, qRT-PCR was conducted in ABI PRISM7900 system (Applied Biosystems, Foster, CA, USA) with microRNA PCR Panel (V3.M) (Exiqon, Danmark), which detected 179 miRNAs in the CSF of 6 depressed and 6 control patients according to the manufacturer’s protocol. Then, the differentially expressed miRNAs from CSF (p<0.05, Fold change >1.5) were further analyzed in serum samples from these patients using qRT- PCR. Finally, the serum differentially altered miRNAs (p<0.05, Fold change >1.5) were further validated by the serum from another 32 depressed patients and 21 healthy individuals using qRT-PCR. Cycling conditions were as follows: 95°C for 10 min and 40 cycles of 15 s at 95°C and 60 s at 60°C. The fluorescence signal was normalized to unify the internal reference level, and the threshold cycle (C_T_) was set in the exponential amplification phase of the PCR. The relative expression levels of miRNAs were calculated based on the number of PCR cycles of miRNAs. Undetectable data were assigned a default C _T_ value of 40. The C_T_ values were normalized according to the delta C _T_ method with the endogenous controls (miR-423–5p) because miR-423–5p was identified as suitable serum-based reference genes of MDD[14], and our results also showed that the C _T_ value of miR-423–5p in both the CSF and the serum between depressed and control subjects are very stable(data no shown). To determine the corresponding ΔC_T_ value, the C_T_ value of the target gene miRNAs was subtracted from the miR-423–5p C_T_ value so to normalize the values. Among the different experimental treatments, the relative expression levels (RELs) of miRNAs were calculated using the following formula: relative gene expression = 2 ^-(ΔC^_T_^of experimental sample - ΔC^_T_^of control sample)^. The data obtained from the miRNA PCR array were analyzed with the GenEx qPCR analysis software.

### Statistics

All statistical analyses were performed using SPSS (version 19; SPSS) and GraphPad Prism (version 5.0, GraphPad Software). Data are presented as mean±SD. Differences in miRNA concentrations between control and MDD subjects were compared using Student’s t-test or Welch’s t-test for equal or unequal variances; the Mann-Whitney U test was used when the distribution was skewed. A difference was considered significant at p < 0.05 or p < 0.01.

## Results

### Patient characteristics

As shown in Table 1, there were 38 depressed patients that had never been treated with antidepressant drugs and 27 control subjects that had no psychiatric disorders. Age and HAMD scores of control and depressed patients in the analysis group were 32.5±9.2 vs 32.0±10.8 (p>0.05) and 4.5±1.2 vs 23.7±4. 8 (t = 9.396, p<0.0001), respectively, there were 2 patients with suicidal ideations among the depressed patients of the analysis group, the control patients of the analysis group there were 4 with renal calculus and 2 with bladder stones. Age and HAMD scores of the control and depressed subjects in the validated group were 35.34±9.5 vs 34.33±10.4 (p>0.05) and 4.4±1.6 vs 25.7± 4.1 (t = 10.168, p<0.0001), respectively, there were 9 patients with suicidal ideations among the depressed patients of the validated group, and the control subjects of the validated group consisted of 21 healthy individuals.

**Table 1:** Characteristics of depressed patients and control subjects.

Variable	Analysis group			Validated group		
	C(n = 6)	T(n = 6)	p-value	C(n = 21)	T(n = 32)	p-value
Sex(M/F)	2/4	3/3	0.925 ^a^	10/11	15/17	0.958 ^a^
Age(years)	32.5±9.2	32.0±9.10.8	0.837 ^b^	35.34±9.5	34.33±10.4	0.720 ^b^
HAMD-scores	4.5±1.2	23.7±4.8	<0.001^b^	4.4±1.6	25.7±4.1	<0.001^b^
Idea of suicide	0/6	2/6	N/A	0/21	9/23	N/A
Relevant diseases	Renal calculus	N/A	N/A	N/A	N/A	N/A
	Bladder stones					

Age and HAMD scores were presented as mean±standard deviation

C group: control subjects with no psychiatric disorders, T group: MDD patients, M: male, F: female

^a^ Pearson Chi-Square;

^b^ Independent sample-t test.

### Measure of RNA concentration and purity about CSF and serum samples

We determined whether miRNAs were detectable in frozen CSF and serum samples collected from depressed and control subjects. For direct measurement of total RNA we used the NanoDrop-1000. RNA concentrations were detected in the CSF specimens (control subjects: 38.39±17.78 ng/μL, depressed patients: 25.05±2.72 ng/μL) and the serum specimens (control subjects: 21.83±5.21ng/μL, depressed patients: 23.73±7.82 ng/μL). The ratio of A 260 to A 280 values (a measure of RNA purity) of all specimens fell in the range of 1.8 to 2.1 (data no shown).

### Detection of differential miRNAs in the CSF and serum of 6 MDD patients

**Fig 1:**
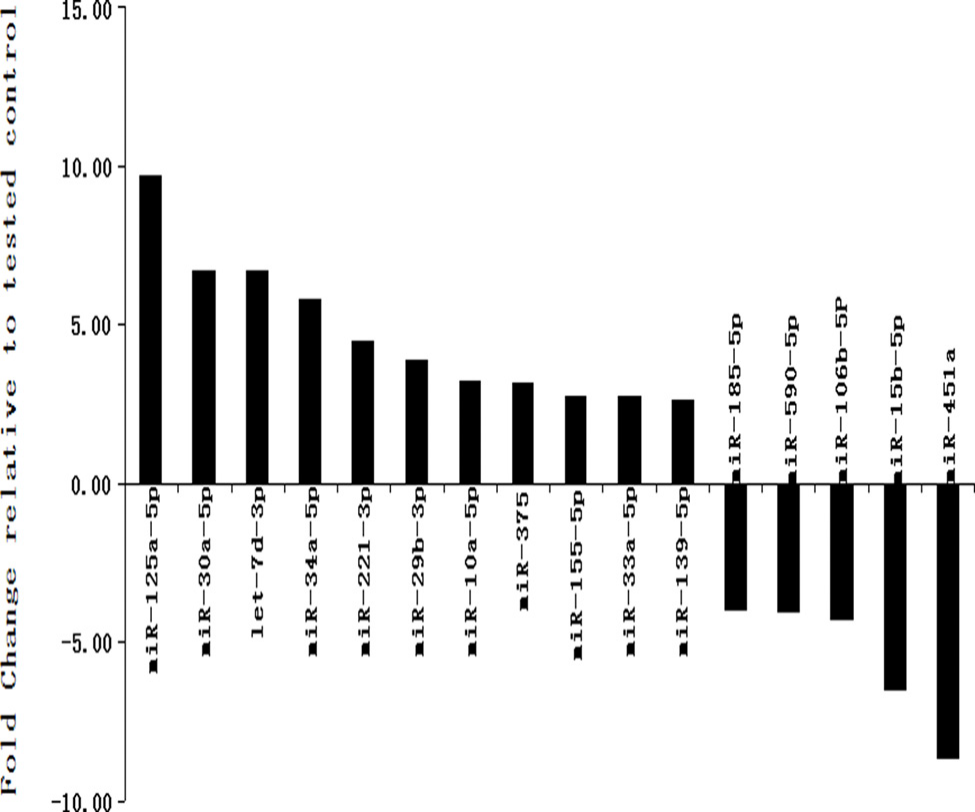
miRNAs that are significantly up-or down-regulated in the CSF from MDD vs. control subjects.

First, the 179 miRNAs in CSF from MDD and control patients were analyzed by microRNA PCR panel analysis. As shown in Table 2, Fig. 1, the results indicated that 16 miRNAs were differentially expressed between control and depressed patients (p<0.05, fold change>1.5), the mean RELs of miR-125a-5p, let-7d-3p, miR-30a-5p, miR-34a-5p, miR-221–3p, miR-29b-3p, miR-10a-5p, miR-375, miR-155–5p, miR-33a-5p and miR-139–5p in the depressed patients were obviously higher than these in the control patients (p<0.05, fold change from 9.75 to 2.63), the mean RELs of miR-451a, miR-15b-5p miR-106b-5p, miR-590–5p and miR-185–5p in the depressed patients were obviously lower than these in the control patients (P<0.05, fold change from-8.64 to-3.99).

**Table 2:** Differentially expressed miRNAs in the CSF between depressed and control subjects.

miRNA ID	MDD patients(n = 6)		Control patients(n = 6)		p value ^a^
	Mean of ΔCt	SD	Mean of ΔCt	SD	
miR-125a-5p	-1.31	1.02	1.96	1.49	0.0012
miR-30a-5p	0.75	1.16	3.69	2.25	0.0143
let-7d-3p	-0.75	1.23	2.19	2.96	0.0422
miR-34a-5p	0.85	1.48	3.38	1.74	0.0411
miR-221-3p	-0.27	0.89	1.89	1.37	0.0092
miR-29b-3p	1.38	1.22	3.52	1.73	0.0223
miR-10a-5p	3.87	0.75	5.94	0.83	0.0249
miR-375	3.92	1.48	5.57	1.06	0.0160
miR-155-5p	3.61	1.29	5.07	0.78	0.0185
miR-33a-5p	3.42	1.39	4.87	0.86	0.0191
miR-139-5p	4.18	1.21	5.57	1.06	0.0375
miR-185-5p	4.15	1.46	2.25	1.75	0.0276
miR-590-5p	3.61	1.29	5.07	1.65	0.0018
miR-106b-5p	1.54	1.12	-0.58	1.99	0.0451
miR-15Bb-5p	2.83	1.16	0.12	0.78	0.0008
miR-451a	-4.3	2.14	-7.42	2.58	0.0455

ΔCt = Ct of target miRNAs-Ct of miR-423-5p(endogenous reference gene)

SD = standard deviation

^a^ Independent sample t test.

**Fig 2:**
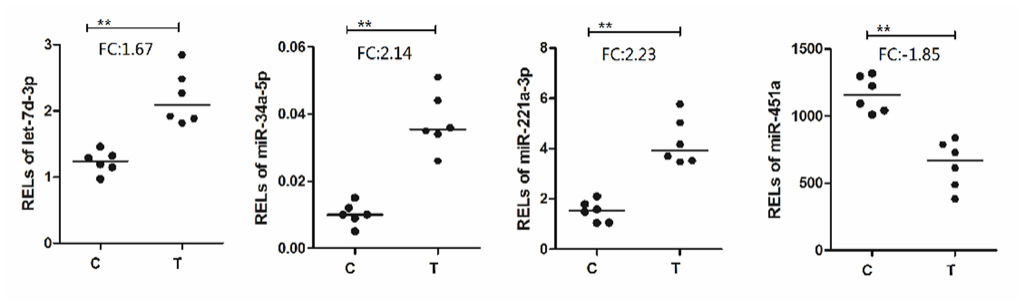
miRNAs that are significantly up- or down-regulated in the serum from the same 6 MDD vs. control subjects. C: control subjects; T: MDD patients; FC: fold change; **p<0.01 vs control.

Then, 16 CSF significantly altered miRNAs were further analyzed in the serum from the same MDD patients using qRT-PCR. As shown in Table 3, Fig. 2, the results indicated that only 4 miRNAs were differentially expressed between control and depressed patients (p<0.05, fold change>1.5), the mean RELs of let-7d-3p, miR-34a-5p and miR-221–3p in the depressed patients were obviously higher than these in the control patients (p<0.01, fold change: 1.67, 2.14, 2.23), and the mean RELs of miR-451a in the depressed patients were obviously lower than that in the control patients (P<0.001, fold change: -1.85). The differences in mean RELs of miR-125a-5p, miR-30a-5p, miR-29b-3p and miR-15b-5p were not statistically significant between control and depressed patients (p>0.05).

**Table 3:** Differentially expressed miRNAs in the serum between depressed and control subjects.

miRNA ID	MDD patients(n = 6)		Control patients(n = 6)		p value ^a^
	Mean of ΔCt	SD	Mean of ΔCt	SD	
miR-125a-5p	0.27	0.88	0.58	0.92	0.5614
miR-30a-5p	7.33	0.81	7.63	0.42	0.4367
let-7d-3p	-0.95	0.46	-0.28	0.28	0.0094
miR-34a-5p	5.61	0.49	6.71	0.52	0.0038
miR-221–3p	-1.73	0.26	-0.55	0.40	0.0001
miR-29b-3p	-2.23	0.17	-2.08	0.27	0.2685
miR-10a-5p	5.11	0.38	5.44	0.52	0.2475
miR-375	4.62	0.63	4.94	0.58	0.3760
miR-155–5p	5.78	0.82	6.61	0.96	0.1427
miR-33a-5p	6.44	0.74	6.94	0.41	0.1857
miR-139–5p	2.95	0.42	3.10	0.51	0.5698
miR-185–5p	3.61	0.42	3.77	0.30	0.4685
miR-590–5p	3.15	0.55	3.24	0.51	0.7703
miR-106b-5p	4.61	0.76	4.88	0.47	0.4741
miR-15Bb-5p	-1.14	0.26	-0.85	0.47	0.2101
miR-451a	-9.51	0.21	-10.18	0.17	0.0001

ΔCt = Ct of target miRNAs-Ct of miR-423–5p(endogenous reference gene)

SD = standard deviation

^a^ Independent sample t test.

### Validation of miR-34a-5p, miR-221-3p, let-7d-3p and miR-451a in the serum of another 32 MDD patients

**Fig 3:**
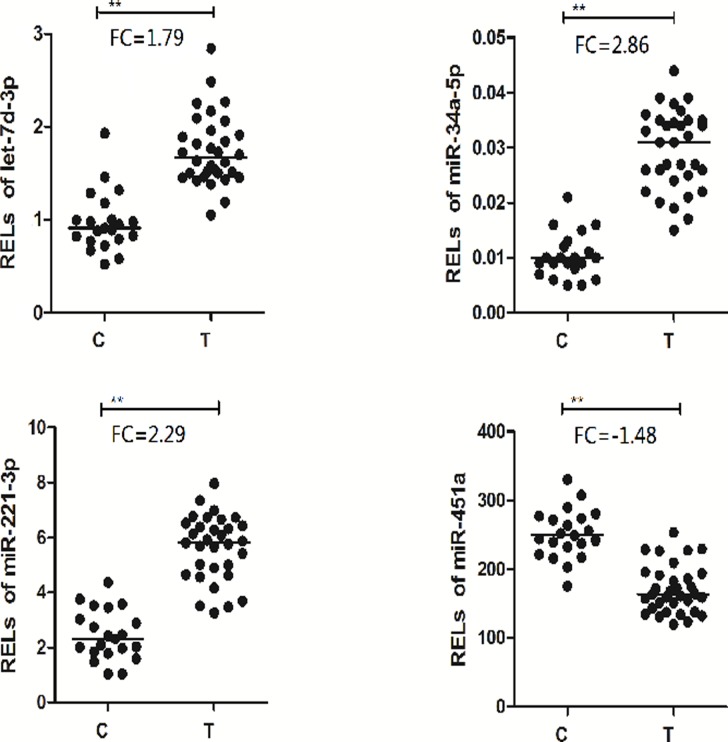
miRNAs that are significantly up-or down-regulated in the serum from another 32 MDD vs. 21 control subjects. C: control subjects, T: MDD patients; FC: fold change; **p<0.0001 vs control.

Four miRNAs (miR-34a-5p, miR-221-3p, let-7d-3p and miR-451a) were significantly altered in serum and were further analyzed in the serum from another 32 MDD patients using qRT-PCR. As shown in Fig. 3, the results indicated that the mean RELs of let-7d-3p, miR-34a-5p and miR-221-3p in the depressed patients were also obviously higher than these in the control subjects (p<0.0001, fold change: 1.79, 2.86, 2.29), the mean RELs of miR-451a in the depressed patients were obviously lower than that in the control subjects (p<0.0001, fold change: -1.48).

### Receiver operating characteristic (ROC) analysis of let-7d-3p, miR-34a-5p, miR-221-3p and miR-451a

**Fig 4:**
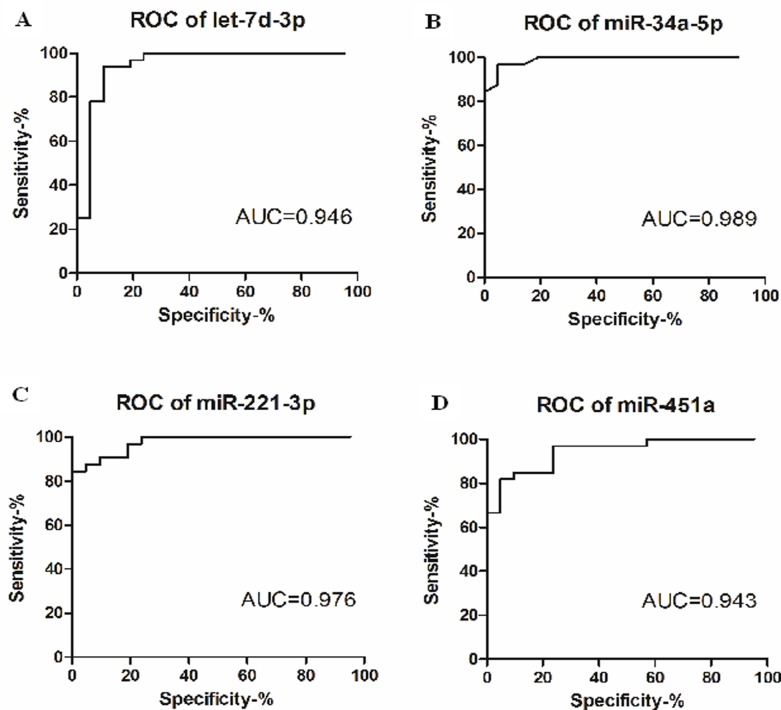
Receiver operating characteristics curve evaluation of serum microRNAs for the diagnosis of MDD. Let-7d-3p (A), miR-34a-5p (B), miR-221-3p (C) and miR-451a (D). AUC: the area under curve.

To observe the relationship between the four microRNAs and MDD, we conducted ROC analysis to estimate the sensitivity and specificity of the diagnostic ability of let-7d-3p, miR-34a-5p, miR-221-3p and miR-451a. As shown in Fig. 4, the ROC curves of let-7d-3p, miR-34a-5p, miR-221-3p and miR-451a reflected obvious separation between MDD and control groups, with an AUC of 0.94[95% confidence interval (CI) = 0.87~1.01, P<0.0001], 0.98 (95% CI = 0.97~1.00, P<0.0001), 0.97 (95% CI = 0.94~1.01, P<0.0001) and 0.94(95% CI = 0.88~1.00, P<0.0001). The specificity of let-7d-3p, miR-34a-5p, miR-221-3p and miR-451a were 90.48%, 95.24%, 90.48% and 90.48%, respectively. The sensitivity of let-7d-3p, miR-34a-5p, miR-221-3p and miR-451a were 93.75%, 96.88%, 90.63% and 84.85%, respectively. let-7d-3p, miR-34a-5p, miR-221-3p and miR-451a displayed a very high sensitivity and specificity for diagnosis of MDD.

### Prediction of target genes and pathway of let-7d-3p, miR-34a-5p, miR-221-3p and miR-451a

To investigate the target genes and pathway of let-7d-3p, miR-34a-5p, miR-221-3p and miR-451a in MDD, target genes were predicted by using miRNA target prediction databases(Target Scan, miRanda and PicTar). The possible regulatory pathway of the target genes were analyzed based on functional annotation according to KEGG pathway terms [15]. The analysis found that some important genes related to MDD, such as serine protein kinases(AKT)[16], serotonin receptors(5HT2A, HTR2C)[17], corticotrophin-releasing hormone receptor (Crhr1)[18], glutamate transporters (SCL1A2)[19], were targeted by miR-451a, miR-221-3p or/ and miR-34a-5p(data no shown). The analysis of regulatory pathway of these target genes indicated a significant enrichment in several pathways related to neuronal brain function of the depression such as PI3K-Akt signaling pathway, Axon guidance, Wnt signaling pathway, Neurotrophin signaling pathway, Hippo signaling pathway, mTOR signaling pathway, ErbB signaling pathway, Cell cycle, Apoptosis, Long-term depression(data no shown).

## Discussion

Our results showed that CSF miRNAs from MDD patients could be detected. Unfortunately, it is very difficult to obtain CSF from MDD patients by lumber puncture. It is more convenient and easier to use serum specific miRNAs from MDD patients for diagnosis. Therefore, we should seriously consider how to look for serum specific miRNAs as biomarker for MDD because the change of CSF miRNAs was poorly related to the alteration of serum miRNAs for CNS diseases[13]. Serum miRNAs that are consistent with CSF changes of miRNAs should be identified in MDD patients, and these miRNAs from CSF should have relative high serum levels and be easily detected in serum. Therefore, Focus 179 microRNA PCR Panels were chosen for the analysis of CSF miRNAs from MDD patients (http://www.Exiqon.com/serum-plasma-miRNA-qPCR). The reasons why we only measured 179 miRNAs instead of performing a microarray for miRNome profiling analysis are as follows: (1) The amount of samples such as serum or CSF is really small, which means that a more sensitive and accurate platform is needed. (2) Not all microRNAs could be detected in serum, and we cared about those which may exist in serum. Exiqon has extensive in-house knowledge of microRNA profiling in serum. All 179 miRNA assays have been carefully selected based on Exiqon’s vast number of in-house analyses of miRNA expression in blood serum and plasma samples as well as on the limited number of peer-reviewed published papers available.

Emerging evidence has demonstrated that dysregulation of miRNAs may contribute to the etiology and pathophysiology of MDD [20]. miRNAs in diseased cerebral tissues are able to be released into CSF, and CSF miRNAs can be used as novel biomarkers for the diagnosis of gliomas [21], but the change in CSF miRNAs from MDD patients is unclear. To our knowledge, this is the first comparison of deregulated miRNAs expression levels in CSF samples from MDD patients who are not being treated with antidepressants compared to that of control patients. The quantitative expression analysis indicated that different expression levels of 16 miRNAs in the CSF were modulated by the MDD patients, and included 11 up-regulated miRNAs (miR-125a-5p, let-7d-3p, miR-30a-5p, miR-34a-5p, miR-221-3p, miR-29b-3p, miR-10a-5p, miR-375, miR-155–5p, miR-33a-5p and miR-139–5p) and 5 down-regulated miRNAs (miR-451a, miR-15b-5p, miR-106b-5p, miR-590–5p and miR-185–5p). After we further analyzed the REL of CSF 16 differential miRNAs in the each patient, we found that there was a obvious high level in the REL of let-7d-3p, miR-29a-3p, miR-30a-5p, miR-34a-5p, miR125a-5p, miR-221-3p. Interesting, the high value came from a patient with suicidal ideation and high HAMD(32 score), however, there were no impact on the the REL of the other 10 differential miRNAs in the same patient(no data shown), and the phenomenon were not found in the addition a patient with suicidal ideation (no data shown) and the serum of MDD patients. Due to the small ample size, whether suicidal ideations with depressed patients have an impact on the CSF results should be investigated in future study. Other studies have shown that the expression of miR-30a-5p, miR-34a-5p, miR-221 and miR-34a were downregulated by antidepressant medications [22–25]. The expression of BDNF was downregulated by the overexpression of miR-34a, miR-30a-5p, let-7d, miR-10a-5p, miR-375 and miR-155 in neurons or brain tissues [26–31], and the low expression levels of BDNF in brain tissue were thought to be part of the main pathogenesis of MDD [32]. The low expression of SIRT1 gene plays an important role in the pathogenesis of MDD[33], studies have found that the overexpression of miR-10a-5p and miR-34a can downregulate the expression of SIRT1 gene [28,34]. miR-185 has also been suggested to participate in the pathogenesis of major depression and/or suicide[35]. Therefore, we believe that the differential change of miRNAs in CSF from MDD patients was involved in the onset and development of MDD. The hypothesis needs to be further validated in depression models in future studies.

We further analyzed serum relative levels of the 16 differentially altered miRNAs from CSF in the same MDD patients. The results showed that the serum relative levels of only 3 miRNAs (miR-34a-5p, miR-221-3p and let-7d-3p) in MDD patients were obviously higher than these in control patients, and the serum relative levels of only miR-451a in MDD patients were obviously lower than that in control patients. miRNAs levels in CSF and serum samples were poorly correlated, and our results are consistent with a recent study by Gallego et al[13], who found that miRNAs from CSF were not strongly correlated to miRNAs from whole blood in psychotic disorders. The mechanisms involved in the obvious difference between CSF and serum are still unclear, and the mechanisms should be investigated in future study. In addition, the serum changes of miR-34a-5p, miR-221-3p, let-7d-3p and miR-451a have further been demonstrated in serum from another 32 MDD patients. By receiver operating characteristic (ROC) curve analysis, let-7d-3p, miR-34a-5p, miR-221-3p and miR-451a displayed a very high sensitivity and specificity for diagnosis of MDD. In addition, our analysis of target genes and pathway of the altered miRNAs showed some important genes related to MDD, such as serine protein kinases(AKT)[16], serotonin receptors(HTR2C)[17], corticotrophin-releasing hormone receptor (CRHR1)[18], glutamate transporters (SCL1A2)[19], were targeted by miR-451a, miR-221-3p or/ and miR-34a-5p (data no shown) and several pathway related to neuronal brain function of the depression, such as PI3K-Akt signaling pathway, Axon guidance, Wnt signaling pathway, Neurotrophin signaling pathway, Hippo signaling pathway, mTOR signaling pathway, ErbB signaling pathway, Cell cycle, Apoptosis, Long-term depression, were significantly enriched by miR-451a, let-7d-3p, miR-221-3p or/ and miR-34a-5p (data no shown). We plan to investigate these target genes or proteins regulated by these altered miRNA and analyze the relationship between these target proteins and MDD in future study. Therefore, our results indicated that the four serum miRNAs might be able to serve as candidate biomarkers for MDD diagnosis, and some researches found that serum miR-221 and miR-34a levels were decreased by antidepressant treatment [15,19]. Although the sample size of our study was not very large, and we did not include other neurodegenerative disorders, such as schizophrenia and anxiety disorder, which may cause restriction in developing MDD biomarkers, our present results could provide the rationale for future investigations of serum and CSF miRNAs for diagnostic and prognostic purposes in MDD. Therefore, a larger sample size and comprehensive study in human should be conducted to further demonstrate the diagnostic ability of the four miRNAs for MDD in future study.

In summary, in this study we have identified that differentially altered miRNAs in CSF might be related to MDD, and serum miR-221-3p, miR-34a-5p, let-7d-3p, and miR-451a might be able to serve as biomarkers for MDD.
